# Snake Bite-Induced Leukoencephalopathy: A Rare Case

**DOI:** 10.7759/cureus.55116

**Published:** 2024-02-28

**Authors:** Vibha Sharma, Sailendra Nayak, Sidharth S Pattnaik, Ambika P Mohanty, Shubhransu Patro

**Affiliations:** 1 Department of General Medicine, Kalinga Institute of Medical Sciences, Kalinga Institute of Industrial Technology, Bhubaneswar, IND

**Keywords:** leukoencephalopathy, neurotoxic, viper, toxin, snake bite

## Abstract

Across the globe, snake envenomation causes significant morbidity and mortality. Although many clinical presentations and complications are observed in different types of snake bites, the incidence of leukoencephalopathy is rare. Although most cases of leukoencephalopathy are seen in viper bites, they are rarely seen in neurotoxic snake bites. In this report, we present a unique case of snake bite-induced leukoencephalopathy following a neurotoxic snake bite. The case highlights the importance of considering this rare complication in cases of snake bites presenting with neurological symptoms, particularly in those affecting higher mental functions.

## Introduction

Snake bites represent a critical and time-sensitive medical emergency, posing a substantial risk of mortality. This preventable public health threat is particularly prevalent in rural areas of tropical and subtropical regions with high rainfall and humid climates. India has the unfortunate distinction of having the highest incidence of snake bite-related deaths globally, with an estimated 35,000-50,000 fatalities annually, as reported by the World Health Organization [1]. Common clinical characteristics of viper bite envenoming include local pain and tissue damage characterized by swelling, blistering, bleeding, and necrosis at the bite site; coagulopathy; and platelet dysfunction, leading to spontaneous systemic hemorrhages and persistent bleeding from fang marks, wounds, or gums. Intracranial bleeding, including anterior pituitary hemorrhage, and multiorgan failure are common causes of death. Specifically, Russell’s viper can cause acute renal failure and neurotoxicity, as has been shown in several studies conducted in South India [2,3].

Neurological manifestations in the form of cerebrovascular complications such as ischemic strokes, hemorrhagic strokes, multiple lobar hemorrhages, subarachnoid and subdural hemorrhages, cerebellar hemorrhage, optic neuritis, delayed cerebellar ataxia, and disseminated encephalomyelitis have been documented, which are predominantly associated with viper bites according to published literature. Although neurological involvement is seen in neurotoxic snake bites, cerebral involvement is rarely reported. Hence, we report a case of neurotoxic snake bite-induced leukoencephalopathy.

## Case presentation

A 60-year-old male laborer by occupation presented to the emergency department (ED) of a tertiary care hospital in the eastern part of India with ptosis, dyspnea, dysarthria, and altered sensorium two hours before presentation, with a history of neurotoxic snake bite (Indian cobra bite) six hours before that. He had no history of exposure to any environmental toxins. He was neither habituated nor addicted to any substances. Medical attention was initially sought at a local hospital, where fang marks were observed on the plantar aspect of the right great toe. The patient received polyvalent anti-snake venom (ASV) but experienced deterioration, leading to referral to our center.

Upon arrival to the ED, the patient was irritable, afebrile, and had tachycardia and tachypnea with normal blood pressure, with an oxygen saturation (SpO_2_) of 96% in room air. The patient’s attendees reported no history of exposure to toxins or drugs or any alleged substance abuse. On further examination, no signs of inflammation were noted at the site of the bite. On systemic examination, bilateral ptosis, equal but sluggishly reacting pupils on either side, diminished deep tendon reflexes, and bilateral extensor plantar response without any signs of meningeal irritation were observed. Other system examinations were unremarkable, and the patient’s bowel and bladder movements were normal.

Based on the patient’s presentation, a provisional diagnosis of a neurotoxic snake bite with encephalopathy was made, prompting the collection of samples for necessary investigations. The patient’s whole blood clotting test (WBCT) was negative, and his arterial blood gas analysis was suggestive of mixed acidosis, necessitating his transfer to the intensive care unit for invasive ventilation. Subsequent investigations revealed no evidence of organ dysfunction and repeated WBCTs yielded negative results.

The patient received polyvalent ASV and other supportive measures. On the third day of hospitalization, his clinical condition started to improve, and he was extubated and subsequently transferred to the high-dependency unit for further management. However, on the seventh day of hospitalization, he experienced a convulsive episode accompanied by a decrease in consciousness level. Consequently, the patient was reintubated and again placed on invasive ventilator support, along with other supportive measures, including antiepileptics and cerebrospinal fluid (CSF) analysis, and magnetic resonance imaging (MRI) of the brain was planned considering a differential diagnosis of metabolic encephalopathy, hypoxic-ischemic encephalopathy, and toxic leukoencephalopathy.

**Figure 1:**
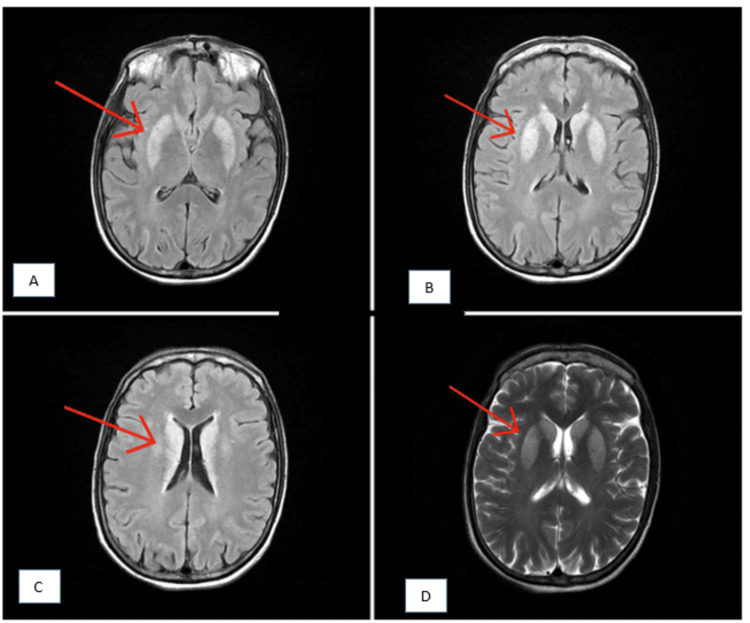
T2-weighted/fluid-attenuated inversion recovery magnetic resonance imaging of the brain showing (A) bilateral symmetrical hyperintensity in the lentiform nucleus, (B) bilateral symmetrical hyperintensity in the corpus striatum, (C) periventricular white matter hyperintensity, and (D) bilateral symmetrical hyperintensity in the corpus striatum.

The results of the CSF analysis were normal, including regular cytological and biochemical parameters, and the patient tested negative for viral markers, specifically herpes simplex and Japanese encephalitis. The MRI of the brain showed bilateral symmetrical T2/fluid-attenuated inversion recovery (FLAIR) hyperintensity in the bilateral basal ganglia and cortex of the perirolandic region, along with a few areas of diffusion restriction (Figure 1).

The MRI of the brain showed hyperintense signal changes in the bilateral basal ganglia and corpus striatum in T2-weighted/FLAIR imaging suggestive of a toxic etiology, likely attributed to the sequelae of the snake bite. Based on the patient’s clinical history and MRI findings, we arrived at a diagnosis of snake bite-induced toxic encephalopathy. As the patient required ventilation support, he underwent a tracheostomy, and an indwelling urinary catheter and a nasogastric tube were placed. On the 14th day of hospitalization, the patient was discharged in a stable clinical condition with advice to follow up at regular intervals. On subsequent visits, the patient’s clinical condition had improved.

## Discussion

Neurological manifestations following venomous snake bites typically stem from the toxic effects of the venom, which include anticoagulant/procoagulant activity and neurotoxicity. Neurological symptoms, though rare, include ptosis, ophthalmoplegia, limb weakness, respiratory failure, palatal weakness, and neck muscle weakness. Cerebrovascular complications such as ischemic strokes, hemorrhagic strokes, multiple lobar hemorrhages, subarachnoid and subdural hemorrhages, cerebellar hemorrhage, optic neuritis, delayed cerebellar ataxia, and disseminated encephalomyelitis have been documented. The proposed etiopathogenesis involves the presence of venom toxins causing hypercoagulability and endothelial damage, immune-mediated vasculitis, and systemic hypotension. The most common and serious complication of the central nervous system following vasculotoxic snake bite is intracranial hemorrhage [4,5]. Although the above-mentioned neurological complications are predominantly associated with viper bites according to published literature, very few cases of neurotoxic snake bite-induced leukoencephalopathy have been reported. Our case was one of neurotoxic snake bite presenting with clinical features consistent with leukoencephalopathy.

The neuroimaging findings in our case suggested toxin-induced leukoencephalopathy rather than hypoxic injury. Although hypoxic-ischemic injury typically involves metabolically active areas (e.g., cortex, basal ganglia, and thalamus), our case deviated from this trend, suggestive of snake bite toxin-induced leukoencephalopathy [6,7]. Therefore, each case of snake bite should be thoroughly evaluated to achieve the best possible outcome for the victim. In rural areas where snake bites are prevalent, cases may not always reach a tertiary center for imaging, potentially contributing to the historical oversight of this entity.

The primary treatment approach for suspected snake envenomation continues to be the use of intravenous polyvalent ASV, recommended empirically. The potential efficacy of ASV is noteworthy, as it may reverse systemic envenomation even after several days or, in cases involving hemostatic abnormalities, two or more weeks. However, it is crucial to administer antivenom promptly in instances of local envenoming without systemic involvement, as its effectiveness diminishes after the initial hours post-bite.

## Conclusions

Snake bite-induced leukoencephalopathy is a rare neurological manifestation of snake bite, particularly in cases of neurotoxic snake bite. Hence, any case of snake bite attending to the ED with features suggestive of involvement of higher mental functions should promptly consider this clinical condition. ASV remains the primary therapy for suspected snake envenomation and should be administered intravenously as early as possible. A timely diagnosis with a high index of suspicion and imaging studies (e.g., MRI of the brain) help to improve management and prognostication.
